# Supplementary material for: Structural and functional analysis of human pannexin 2 channel
Source: Nat Commun. 2023 Mar 27;14:1712. doi: 10.1038/s41467-023-37413-z (PMC10043284; doi:10.1038/s41467-023-37413-z)
Supplement: Supplementary file 1 — Supplementary Information [file 41467_2023_37413_MOESM1_ESM.pdf]

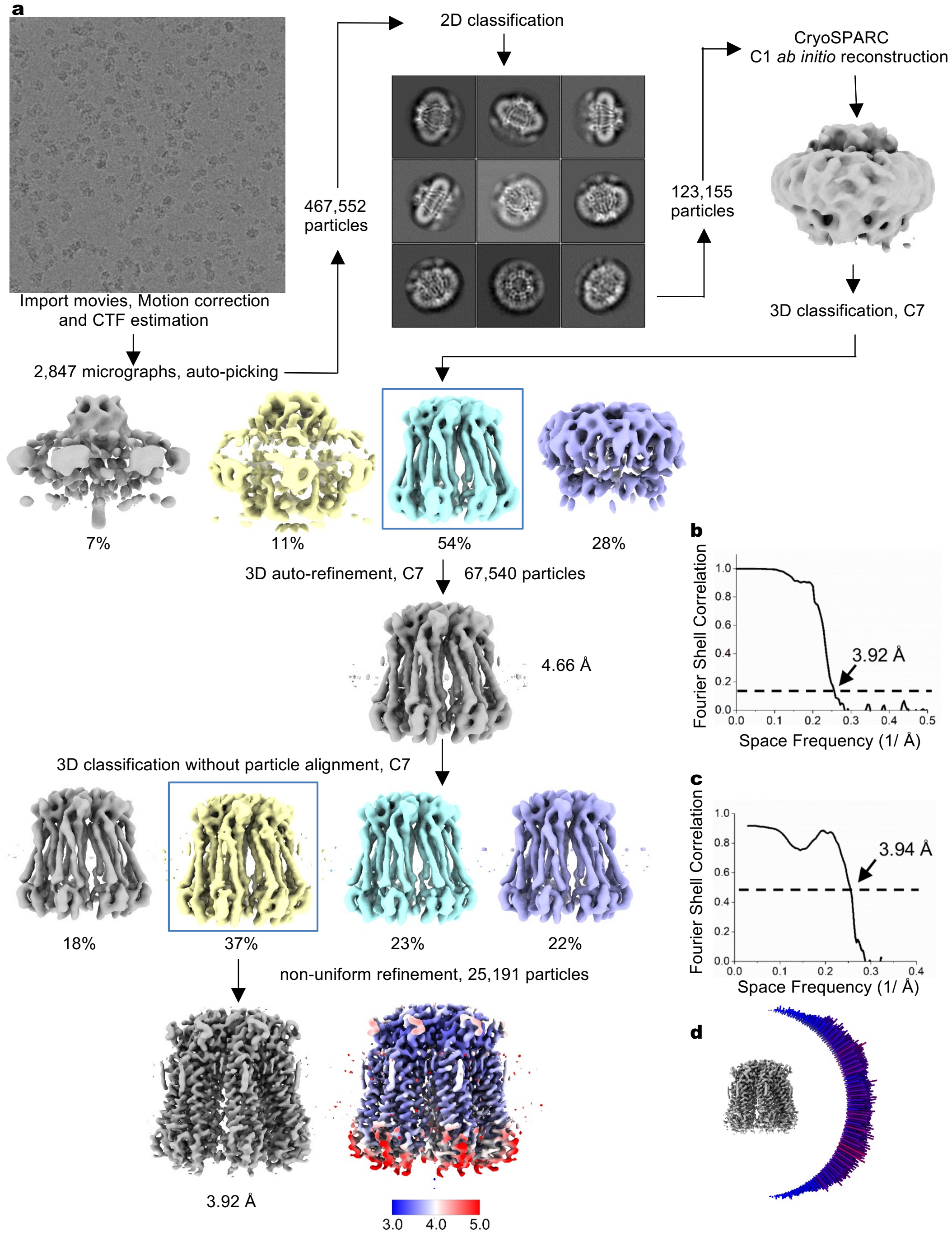

Supplementary Fig. 1 | Cryo-EM reconstruction of human PANX2. a Flowchart of image processing in RELION and CryoSPARC. Representative micrograph and 2D classes are shown. Also shown is the final reconstruction colored by local resolution. **b** Fourier shell correlations (FSC) between the two independent half maps following non-uniform refinement. The dotted line indicates a threshold of 0.143. **c** FSC between the refined model and the full map following real space refinement. **d** Orientation plot of particles used in the final reconstruction.

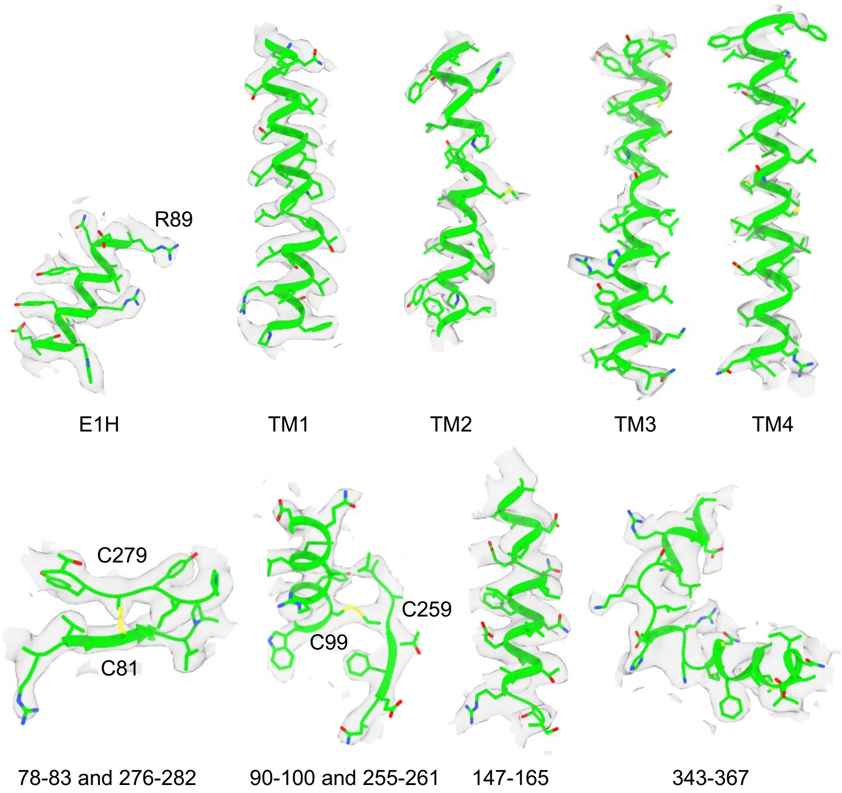

Supplementary Fig. 2 | Cryo-EM density. Segments of the final refined model and the corresponding cryo-EM densities are shown. The transmembrane helices and disulfide bonds are highlighted.

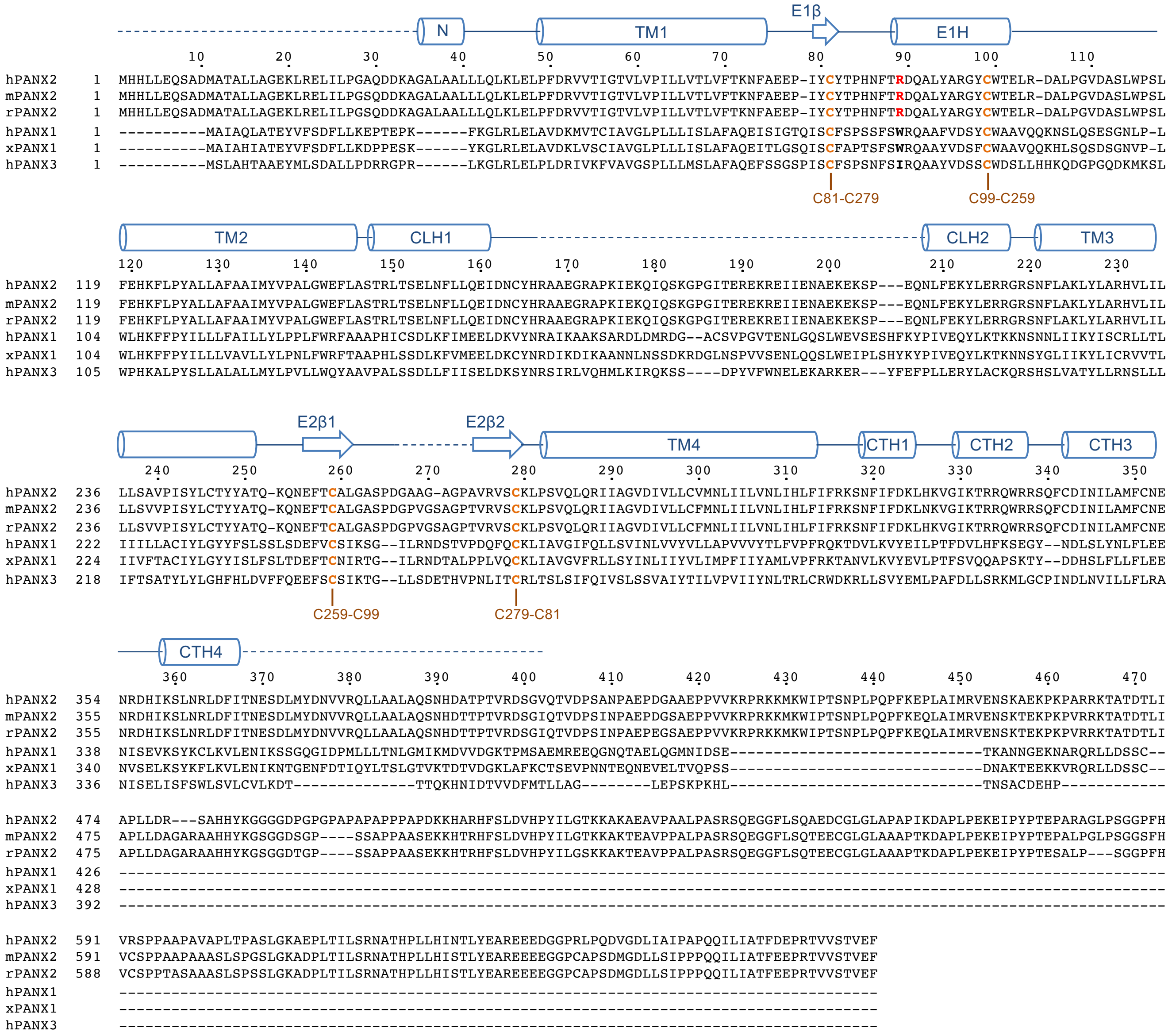

Supplementary Fig. 3 | Sequence alignment of pannexin channels. Aligned protein sequences include *Homo sapiens* PANX2 (hPANX2, NCBI sequence: NP_443071.2), *Mus musculus* PANX2 (mPANX2, NCBI sequence: NP_001002005.2), *Rattus norvegicus* PANX2 (rPANX2, NCBI sequence: NP_955441.2), *Homo sapiens* PANX1 (hPANX1, NCBI sequence: NP_056183.2), *Xenopus tropicalis* PANX1 (xPANX1, NCBI sequence: NP_001123728.1), and *Homo sapiens* PANX3 (hPANX3, NCBI sequence: NP_443191.1). Secondary structure elements on the basis of hPANX2 are indicated above the sequences, and unstructured regions are indicated by dashed lines. Amino acids critical for channel function are highlighted in colors.

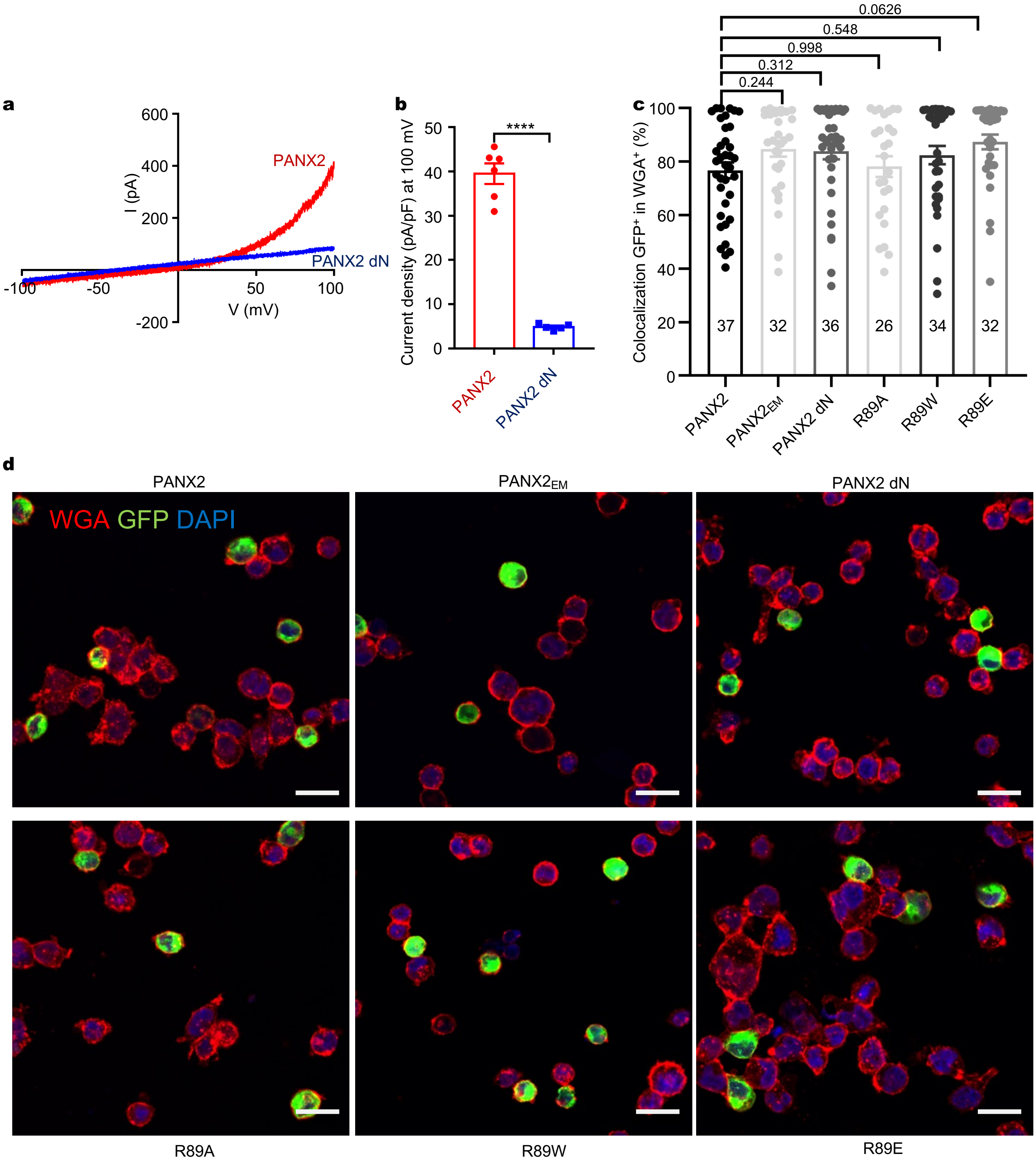

Supplementary Fig. 4 | Function and membrane expression of human PANX2 and mutants. a Representative current-voltage curves for the wild-type full-length human (PANX2) and N-terminally truncated (PANX2 dN) channels expressed in HEK293T cells. **b** Current densities for the full-length and N-terminally truncated human PANX2 channels. Bars represent mean ± SEM (n = 6 and 5 independent cells for the wild type and dN mutant, respectively; **** indicates $p < 0.0001$). **c** Surface expression of the wild-type PANX2 and mutants assessed by immunofluorescence staining. Bars represent mean ± SEM. *p* values are indicated (one-way ANOVA followed by a Tukey-Kramer post-hoc test, numbers of independent cells are indicated). **d** Representative images of immunofluorescent staining of the wild-type human PANX2 and mutants (Red, wheat germ agglutinin; Green, GFP; Blue, DAPI). Scale bar = 20 μm.

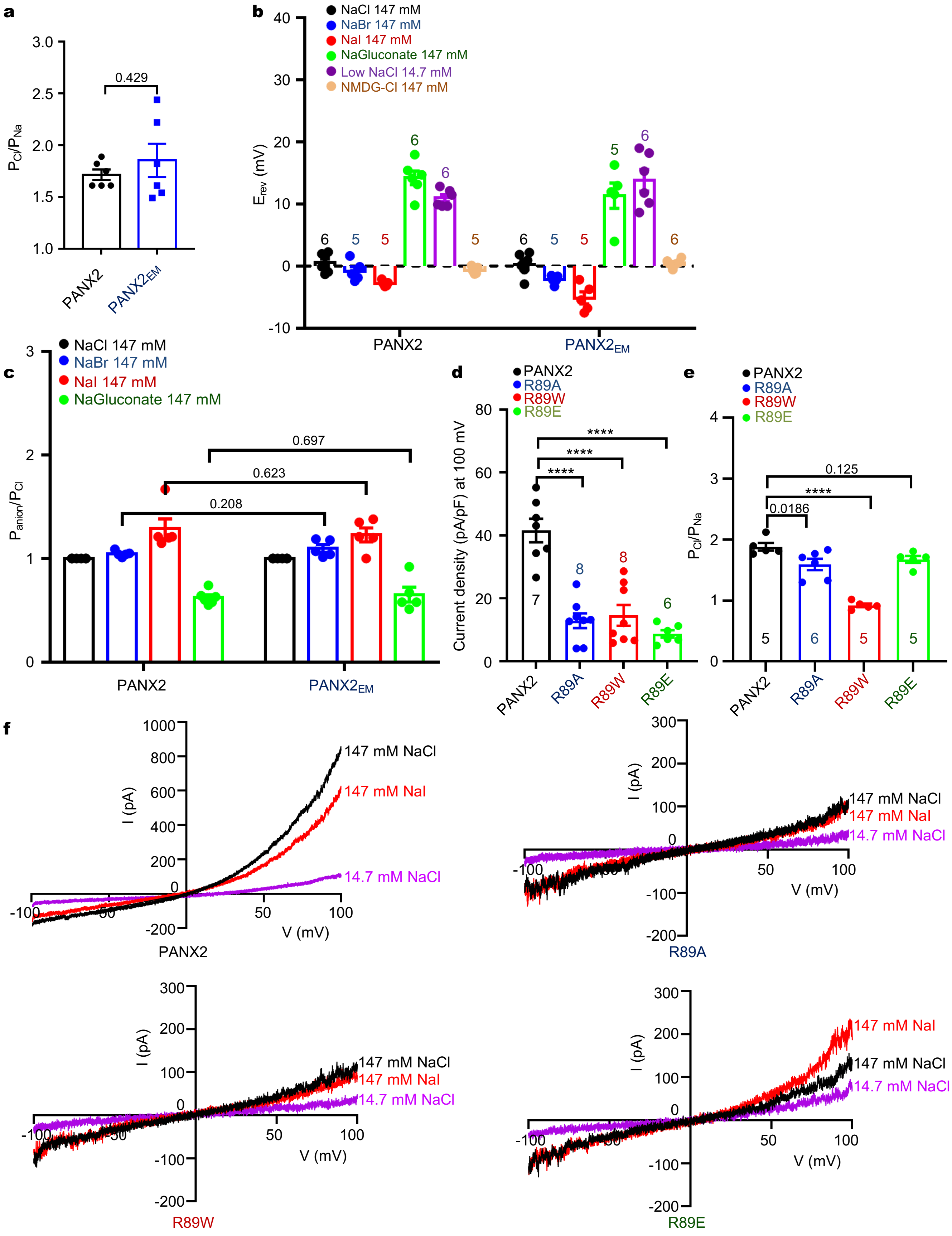
a
3.0
2.5
2.0
1.5
1.0
P_{Cl}/P_{Na}
0.429
PANX2
$PANX2_{EM}$
b
NaCl 147 mM
NaBr 147 mM
NaI 147 mM
NaGluconate 147 mM
Low NaCl 14.7 mM
NMDG-Cl 147 mM
40
30
20
10
0
-10
E_{rev} (mV)
6
5
5
6
6
5
6
5
5
5
6
6
PANX2
$PANX2_{EM}$
c
NaCl 147 mM
NaBr 147 mM
NaI 147 mM
NaGluconate 147 mM
3
2
1
0
P_{anion}/P_{Cl}
0.697
0.623
0.208
PANX2
$PANX2_{EM}$
d
PANX2
R89A
R89W
R89E
80
60
40
20
0
Current density (pA/pF) at 100 mV
****
****
****
7
8
8
6
PANX2
R89A
R89W
R89E
e
PANX2
R89A
R89W
R89E
4
3
2
1
0
P_{Cl}/P_{Na}
0.125
****
0.0186
5
6
5
5
PANX2
R89A
R89W
R89E
f
1000
800
600
400
200
0
-200
I (pA)
-100
-50
50
100
V (mV)
147 mM NaCl
147 mM NaI
14.7 mM NaCl
PANX2
300
200
100
0
-100
-200
I (pA)
-100
-50
50
100
V (mV)
147 mM NaCl
147 mM NaI
14.7 mM NaCl
R89A
300
200
100
0
-100
-200
I (pA)
-100
-50
50
100
V (mV)
147 mM NaCl
147 mM NaI
14.7 mM NaCl
R89W
300
200
100
0
-100
-200
I (pA)
-100
-50
50
100
V (mV)
147 mM NaI
147 mM NaCl
14.7 mM NaCl
R89E

Supplementary Fig. 5 | Ion selectivity of wild-type and mutant PANX2 channels. a Permeability ratios (P_{Cl}/P_{Na}) of the wild-type human PANX2 and truncated construct $PANX2_{EM}$ (mean ± SEM, n = 6 independent cells). *p* value is indicated (unpaired two-tailed t-test). **b** Reversal potential measurements of human PANX2 and $PANX2_{EM}$ recorded in external solutions with different concentrations of cations and anions (mean ± SEM, numbers of independent cells are indicated). **c** Anion permeability ratios (P_{anion}/P_{Cl}) for human PANX2 and $PANX2_{EM}$ (mean ± SEM, n = 5 independent cells, unpaired two-tailed t-test). **d** Current densities of the wild-type human PANX2 and point mutants (R89A, R89W, and R89E) recorded in symmetrical NaCl conditions (mean ± SEM, numbers of independent cells are indicated, one-way ANOVA followed by a Tukey-Kramer post-hoc test, **** indicates $p < 0.0001$). The external solution comprised 147 mM NaCl, 10 mM HEPES pH 7.4, 13 mM Glucose, 2 mM KCl, 2 mM $CaCl_2$, 1 mM $MgCl_2$, and the internal solution contained 147 mM NaCl, 10 mM EGTA, 10 mM HEPES pH 7.4. **e** Permeability ratios (P_{Cl}/P_{Na}) of the wild-type human PANX2 and mutants (mean ± SEM, numbers of independent cells are indicated, one-way ANOVA followed by a Tukey-Kramer post-hoc test, **** indicates $p < 0.0001$). Currents were recorded with low NaCl external solution containing 14.7 mM NaCl, 10 mM HEPES pH 7.4, 2 mM Ca-Gluconate, 1 mM Mg-Gluconate, and 245 mM sucrose. The internal solution contained 147 mM NaCl, 10 mM EGTA, 10 mM HEPES pH 7.4. **f** Representative current-voltage traces for the wild-type human PANX2 and mutants (R89A, R89W, and R89E) expressed in HEK293T cells and recorded in different external solutions as indicated.

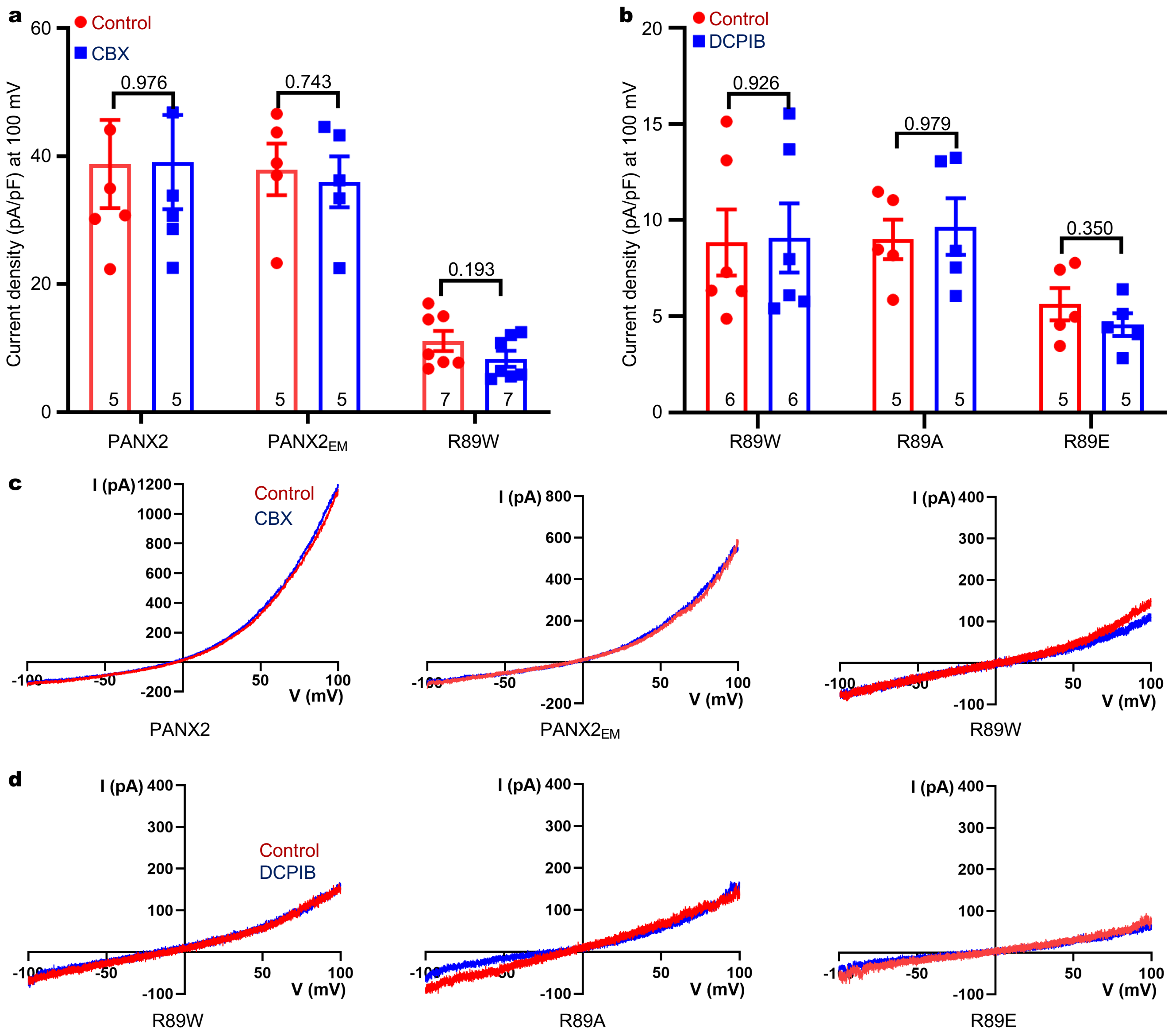

Supplementary Fig. 6 | **Pharmacology of PANX2 and mutants. a** Current densities of human PANX2, $PANX2_{EM}$, and PANX2 R89W in the absence or presence of 0.1 mM CBX (mean ± SEM, numbers of independent cells are indicated, unpaired two-tailed t-test). **b** Current densities of human PANX2 mutants R89W, R89A, and R89E in the absence or presence of 75 μM DCPIB (mean ± SEM, numbers of independent cells are indicated, unpaired two-tailed t-test). **c** Representative current-voltage traces of human PANX2, $PANX2_{EM}$, and PANX2 R89W in the absence (red) and presence (blue) of 0.1 mM CBX. **d** Representative current-voltage traces of human PANX2 mutants, R89W, R89A and R89E, in the absence (red) and presence (blue) of 75 μM DCPIB.

Supplementary Table 1 | Cryo-EM data collection, refinement and validation statistics

	hPANX2<br>(EMDB-28902)<br>(PDB 8F7C)
**Data collection and processing**	
Magnification	75,000
Voltage (kV)	300
Electron exposure ($e^{-}/Å^2$)	49
Defocus range (μm)	-1.0 to -2.4
Pixel size (Å)	0.9
Symmetry imposed	C7
Initial particle images (no.)	467,552
Final particle images (no.)	25,191
Map resolution (Å)	3.92
FSC threshold	0.143
Map resolution range (Å)	3.0-5.0
**Refinement**	
Initial model used (PDB code)	AlphaFold
Model resolution (Å)	3.94
FSC threshold	0.5
Model resolution range (Å)	3.94
Map sharpening *B* factor ($Å^2$)	-219.5
Model composition	
Nonhydrogen atoms	15,925
Protein residues	2,009
Ligands	0
B factors ($Å^2$)	
Protein	78.73
Ligand	N/A
R.m.s. deviations	
Bond lengths (Å)	0.002
Bond angles (°)	0.538
Validation	
MolProbity score	1.41
Clash score	4.39
Poor rotamers (%)	0
Ramachandran plot	
Favored (%)	96.80
Allowed (%)	3.20
Disallowed (%)	0

Supplementary Table 2 | **Primers used for cloning**

Construct	Primer	Sequence (5′-3′)
$PANX2_{EM}$	forward	CTTCATTACTAATGAGAGTGACGCCGACTTGGAAGACAACTGG
	reverse	GTTGTCTTCCAAGTCGGCGTCACTCTCATTAGTAATGAAGTCCAATC
hPANX2 R89A	forward	CACAACTTTACCGCTGATCAAGCATTG
	reverse	CAATGCTTGATCAGCGGTAAAGTTGTG
hPANX2 R89E	forward	CACAACTTTACCGAGGATCAAGCATTG
	reverse	CAATGCTTGATCCTCGGTAAAGTTGTG
hPANX2 R89W	forward	CACAACTTTACCTGGGATCAAGCATTG
	reverse	CAATGCTTGATCCCAGGTAAAGTTGTG
hPANX2 dN	forward	CCGCTCGAGCCACCATGCTGCAATTGAAGTTGGAACTGCC
	reverse	CACAGCGGCGGCAGCAGTTG
